# Evolutionary History of the Cancer Immunity Antigen *MAGE* Gene Family

**DOI:** 10.1371/journal.pone.0020365

**Published:** 2011-06-10

**Authors:** Yukako Katsura, Yoko Satta

**Affiliations:** Department of Evolutionary Studies of Biosystems, The Graduate University for Advanced Studies (Sokendai), Hayama, Kanagawa, Japan; California State University Fullerton, United States of America

## Abstract

The evolutionary mode of a multi-gene family can change over time, depending on the functional differentiation and local genomic environment of family members. In this study, we demonstrate such a change in the melanoma antigen (*MAGE*) gene family on the mammalian X chromosome. The *MAGE* gene family is composed of ten subfamilies that can be categorized into two types. Type I genes are of relatively recent origin, and they encode epitopes for human leukocyte antigen (HLA) in cancer cells. Type II genes are relatively ancient and some of their products are known to be involved in apoptosis or cell proliferation. The evolutionary history of the *MAGE* gene family can be divided into four phases. In phase I, a single-copy state of an ancestral gene and the evolutionarily conserved mode had lasted until the emergence of eutherian mammals. In phase II, eight subfamily ancestors, with the exception for *MAGE-C* and *MAGE-D* subfamilies, were formed via retrotransposition independently. This would coincide with a transposition burst of *LINE* elements at the eutherian radiation. However, *MAGE-C* was generated by gene duplication of *MAGE-A*. Phase III is characterized by extensive gene duplication within each subfamily and in particular the formation of palindromes in the *MAGE-A* subfamily, which occurred in an ancestor of the Catarrhini. Phase IV is characterized by the decay of a palindrome in most Catarrhini, with the exception of humans. Although the palindrome is truncated by frequent deletions in apes and Old World monkeys, it is retained in humans. Here, we argue that this human-specific retention stems from negative selection acting on *MAGE-A* genes encoding epitopes of cancer cells, which preserves their ability to bind to highly divergent HLA molecules. These findings are interpreted with consideration of the biological factors shaping recent human *MAGE-A* genes.

## Introduction

The evolutionary mode of a gene family, namely the process of birth and death of genes and the extents of sequence divergence, depends on the functional divergence of duplicated genes and on the local structure of the genome where the family resides [1], [2]. Here, local structure of the genome refers to tandem or inverted repeats (IRs). The evolution of a gene family on IRs can be particularly complex as a result of homogenization by frequent gene conversion and structural instability such as due to frequent insertions and/or deletions.

Warburton et al. (2004) found a preponderance of large, IRs with a high degree of similarity between repeats on the X and Y chromosomes (∼30% of IRs in the human genome are on the X and Y chromosomes) [3]. Many IRs on the X and Y contain genes expressed predominantly in the testis [3]. Warburton and his colleagues suggested that these IRs play an important role in human genome evolution. However, the precise role of IRs in evolution has remained unclear. Therefore, in this study, we attempt to examine the tempo and mode of gene family evolution in IRs, with a specific focus on the melanoma antigen (*MAGE*) gene family, in which members are located on a large (∼100 kb) palindrome on the human X chromosome.

*MAGE* was originally identified as “a melanoma antigen” and later *MAGE* and its homologs were discovered to form a multi-gene family in eutherian genomes [4]–[7]. *MAGE* homologous sequences have been found in some vertebrates (zebrafish and chicken) [8], [9] and invertebrates (fruit fly) [10]. In the human genome, this family is composed of 10 subfamilies and each subfamily is made up of one to 15 genes [7]. In addition to classification by subfamily, *MAGE* genes can also be classified into type I or type II, based on their expression patterns and function. Type I genes are composed of three subfamilies (*MAGE-A*, to –*C*) and type II genes of seven subfamilies (*MAGE-D* to -*F*, -*H*, -*L2*, *NDN*, *NDNL2*). Type I genes are expressed in highly proliferating cells such as tumors, placenta and germ line cells [4]. Type II genes, by contrast, are ubiquitously expressed in somatic cells, and some type II genes are known to be involved in apoptosis or cell proliferation [11].

All type I *MAGE* genes are located on the X chromosome and encode tumor antigens that play a key role in cancer immunity. Peptides in the human MAGE homology domain (MHD), which is 160–170 amino acid long, are epitopes for human leukocyte antigen (HLA) class I molecules [4]. When the antigen (peptide in the MHD) on a tumor cell binds to a receptor on a killer T-cell, the T-cell attacks the tumor cell [4], [12]. *HLA* is exceptionally polymorphic in the human genome and different *HLA* alleles can bind different epitopes [13], [14]. *MAGE* genes may encode many epitopes so as to bind to, or react with, every HLA molecule. Thus, it is of interest to trace the origin of the association between *HLA* and *MAGE* as well as to determine how the genetic diversity in the epitope-coding region has evolved and been maintained.

Many *MAGE* genes are thought to be mammalian-specific [7]. In addition, most eutherian *MAGE* genes have a single exon to encode a protein and therefore they are likely to have derived from retrotransposition of *MAGE-D* [7], because only *MAGE-D* subfamily members have 14 exons where an ORF is encoded between the second to 12th exons [15]. Yet, the relationship between type I and type II genes has not been fully investigated and the mode of diversification of these genes remains unclear.

In this study, we investigate the evolutionary history of the *MAGE* gene family. First, we searched for the most anciently diverged *MAGE* genes in vertebrate and invertebrate genomes. Second, we investigated how and when ancestors of each three type I and seven type II subfamilies were generated with special reference to their mode of amplification. Third, we focus on the *MAGE*-*A* subfamily (one of the type I subfamilies) and demonstrate how the genome arrangement has occurred in primates. Finally, we show that some human *MAGE*-*A* genes have undergone negative selection against homogenization by gene conversion in order to retain their genetic variations among amino acid sequences. We suggest that this selection is related to the maintenance of a variety of HLA epitopes in cancer cells.

## Materials and Methods

### Sequences used

Human (*Homo sapiens*) nucleotide sequence data and corresponding gene information were obtained from the NCBI database (build 36.3; http://www.ncbi.nlm.nih.gov/). Syntenic or homologous genomic sequences from other primates and mammals, including opossums (*Monodelphis domestica*) and platypuses (*Ornithorhynchus anatinus*), were retrieved from the NCBI and Ensembl databases (http://uswest.ensembl.org/index.html). To find conserved synteny between the human X chromosome and chromosomes in other animals, BLAST analyses using human *MAGE* genes as queries were performed. To identify homologous sequences, we use 70% as a cut-off value for BLAST searches.

### Identification of genomic structures

Identification of IRs and tandem repeats was conducted using a dot-matrix approach [16]. GenomeMatcher [17] was then used to obtain detailed information on nucleotide sequence similarity between duplicate units. A diagram drawn by this program depicts the extent of similarity between sequences using color codes, with red representing similarity greater than 95%, orange representing approximately 90%–95%, green representing approximately 85%–90%, and blue representing lower than 85%.

### Phylogenetic and molecular evolutionary analyses

To study phylogenetic relationships among *MAGE* family members, 158 coding sequences (CDSs) in the human, chimpanzee (*Pan troglodytes*), macaque (*Macaca mulatta*), mouse (*Mus musculus*), cow (*Bos taurus*), dog (*Canis lupus*), opossum, platypus, chicken (*Gallus gallus*) and zebrafish (*Danio rerio*) genome were retrieved from the NCBI database (Table S1). *MAGE* homologs were also searched in Ensembl database of the western African clawed frog (*Xenopus tropicalis*), lampreys (*Petromyzon marinus*), lancelets (*Branchiostoma floridae*), tunicates (*Ciona intestinalis*) and sea urchins (*Strongylocentrotus purpuratus*). For each of these species, we searched for *MAGE* homologs over the whole genomes. In the searches for homologs, *MAGE-D* subfamily members were used as a query, because *MAGE-D* is thought to be the ancestral *MAGE* subfamily [7]. When we use other human *MAGE* sequences as a query, we found that sequences detected were already included in the result obtained using *MAGE-D*.

In the human genome, there were 37 annotated *MAGE* genes on the X chromosome: 15 *MAGE-A*s, 11 *MAGE-B*s, three *MAGE-C*s, five *MAGE-D*s, two *MAGE-E*s and one *MAGE-H*. In addition, two *MAGE-F*s are located on chromosome 3, and *necdin-like 2* (*NDNL2* or *MAGE-G*), *MAGE-like 2* (*MAGE-L2*) and *necdin* (*NDN*) are on chromosome 15. Besides the annotated genes, a homologous sequence (*psMAGEA-like: psMAGEAL*, NC_000023: 2765558‥ 2770471) corresponding to the human *MAGE* pseudogene, *psMAGEA* (NC_000023: complementary 151952946‥151957859), was identified. Gene abbreviations used in this study follow the standards used for human genes.

The sequences obtained were aligned using Clustal W software [18] with manual corrections. The sequences of human *MAGE-H*, -*A5*, and mouse -*A9* were short. These were discarded because inclusion of these sequences made a meaningful sequence alignment short. The number of nucleotide differences per site (*p*-distance) was then calculated using MEGA4 [19], and the phylogeny was constructed using the neighbor-joining (NJ) [20] method available in this software. Phylogenies were also constructed with Randomized A(x)ccelerated Maximum Likelihood (RAxML) [21] and Bayesian (Bayes) methods. A program for the RAxML method is provided by http://phylobench.vital-it.ch/raxml-bb/ and that for the Bayes method is MrBayes 3 [22]. The alignments used here are available upon request. DnaSP v5 [23] was used for the window analysis of nucleotide divergence. RepeatMasker [24] was used to screen sequences for interspersed repeats in the NCBI database. A program, GENECONV [25] was used to detect gene conversion.

### Transcription factor binding sites

Transcription factor binding sites (TFBs) were examined using the TRANSFAC R4.3 database [26], available on the TFBIND website (http://tfbind.ims.u-tokyo.a.c.jp/) [27]. To find a candidate TFB, sequences upstream of target genes were aligned, and highly conserved sequences were chosen. The sequences were checked for the presence of TFBs annotated in the database.

## Results

### Origin of the vertebrate and mammalian *MAGE* gene family

To identify *MAGE* homologs in lampreys, lancelets, tunicates, and sea urchins, a BLAST search was performed for their genome and EST sequences, using *MAGE-D* genes as queries. Although there were no detectable homologous genes in lampreys and sea urchins, hypothetical genes in both tunicates (XM_002119518) and lancelets (XM_002613563) showed 37% sequence similarity with the human *MAGE-D1*. The BLAST search results indicated that the emergence of *MAGE* gene could have occurred before the divergence of Protochordata from Chrodata.

**Figure 1:**
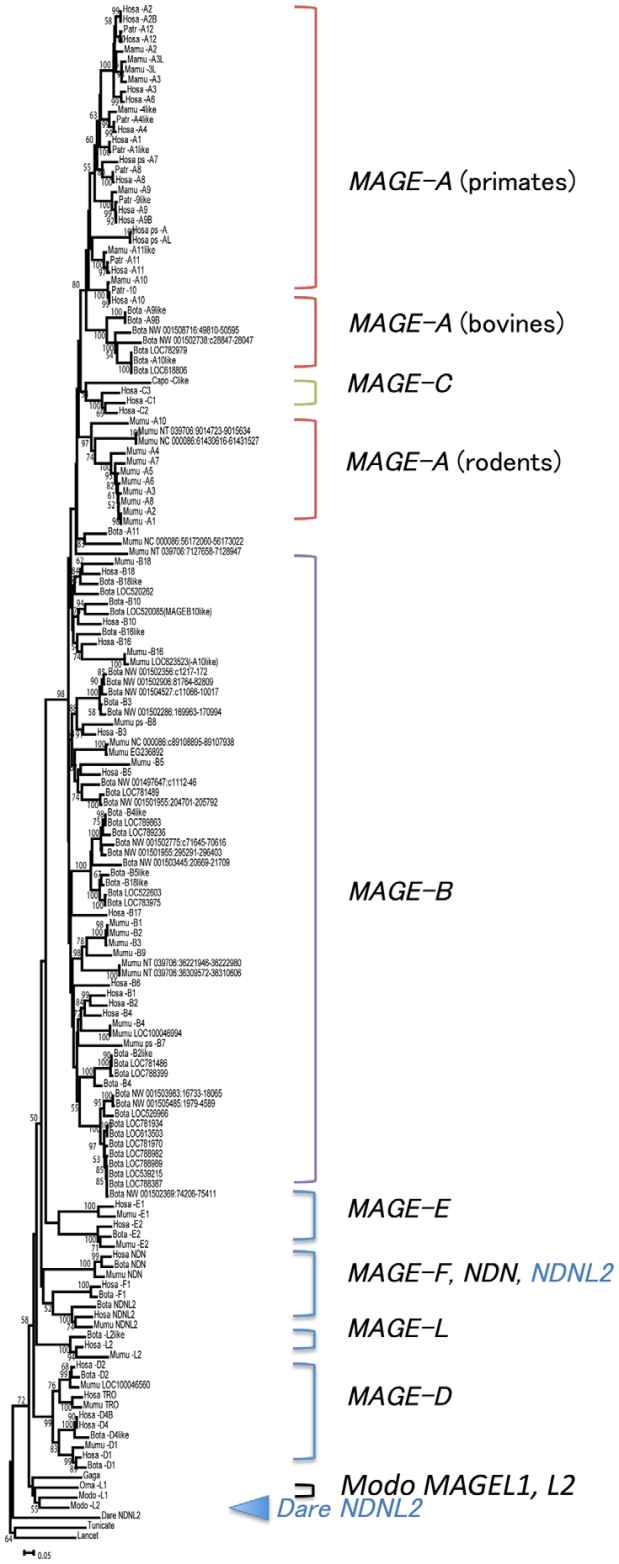
Phylogeny of the *MAGE* gene family. CDSs of 158 *MAGE* genes were used (see Table S1). The CDS compared is 204 bp long. After alignment, all gaps were excluded for tree construction. Subfamily clusters are shown. The number at each node is the bootstrap value supporting the node. Fish *NDNL2* (*Dare NDNL2*) and mammal *NDNL2* are shown in blue. Species name abbreviations are as follows: Bota (*Bos taraus*), Capo (*Cavia porcellus*), Dare (*Danio rerio*), Gaga (*Gallus gallus*), Hosa (*Homo sapiens*), Mamu (*Macaca mulatta*), Modo (*Monodelphis domestica*), Mumu (*Mus musculus*), Orna (*Ornithorhynchus anatinus*), and Patr (*Pan troglodytes*). Figure S1 is an enlarged version of this figure and has legible text.

In jawed vertebrates, the zebrafish genome possesses a single *MAGE* gene, *Necdin-like 2* (*DareNDNL2*) [8]. *NDNL2* genes are found also in humans, mice and cows, but eutherian *NDNL2s* are processed genes and have a single exon, whereas *DareNDNL2* possesses ∼11 exons. A phylogenetic tree based on amino acid sequences shows that eutherian *NDNL2*s are paraphyletic to *DareNDNL2* (Figs. 1 and S1): *DareNDNL2* is a “primary” ortholog of eutherian *MAGE* genes [28]. This phylogenetic relationship (topology of the tree) is also supported by RAxML and Bayes trees (data not shown).

Each of the frog and chicken genomes possesses only one *MAGE* gene. In both cases, concerning the syntenic relationship with *DareNDNL2*, position of the gene on a chromosome could not be confirmed because of the incomplete assignment of genes on chromosomes in these species. However, given that phases at each exon and intron boundary in the CDS of fishes, frogs and chickens were well conserved (Table 1), the single *MAGE* genes in the frog and chicken are likely to be one-to-one orthologs of *DareNDNL2*.

**Table 1:** Phases at exons in the *MAGE* coding sequence of zebrafish, African clawed frog, chicken and mammals.

Exon:^a^	1		2		3		4 (64)		5 (80)		6 (95)		7 (80)		8 (43)		9 (63)		10 (115)		11	
Phase:	S	E	S	E	S	E	S	E	S	E	S	E	S	E	S	E	S	E	S	E	S	E
zebra fish	–	–	–	0	0	0	0	1	1	0	0	2	2	1	1	2	2	2	2	0	0	–
Frog					0	0	0	1	1	0	0	2	2	1	1	2	2	2	2	0	0	0
Chicken	–	–	–	0	0	0	0	1	1	0	0	2	2	1	1	2	2	2	2	0	0	–
Platypus					0	0	0	1	1	0	0	2	2	1	1	2	2	2	2	0	0	0
Opossum	0	0	0	0	0	0	0	1	1	0	0	2	2	1	1	2	2	2	2	0	0	0
human (*D2*)	–	0	0	0	0	0	0	1	1	0	0	2	2	1	1	2	2	2	2	0	0	–
human (*D3*)	–	0	0	0	0	0	0	1	1	0	0	2	2	1	1	2	2	2	2	0	0	–

a : Only protein coding exons are shown. Numbers in parentheses indicate the size of exons that are conserved from fishes to mammals. Exceptions are exon 6 in opossum and human *D3*; exon size is 98 bp and 92 bp, respectively.

Phase information for each species is ENSDART00000081038 for the zebra fish, ENSXETT00000047694 for the frog, DQ983362 for the chicken, NW_001794330 for the platypus, NW_001587054 for the opossum, ENST00000375068 for human *D2*, and ENST00000173898 for human *D3*.

Although only a single *MAGE* was found in fishes, frogs and chickens, humans and mice have multiple subfamilies of *MAGE* genes [7]. Thus, it is interesting to investigate *MAGE* homologs in monotremes (platypus) and marsupials (opossum). A full-genome BLAST search using human *MAGE-D1* as a query detected one *MAGE*-like (*MAGEL*) sequence in the platypus and two *MAGEL*s in the opossum. These were tentatively named *OrnaMAGEL* and *ModoMAGEL1*/*L2*, respectively. BLAST searches using other *MAGE* genes such as *DareNDNL2* as a query resulted in detection of the same genes.

Opossums *ModoMAGEL1* and *ModoMAGEL2* are located on chromosomes X and 8, respectively. *ModoMAGEL2* is coded by a single exon, whereas *ModoMAGEL1* is coded by 11 exons. Thus, *ModoMAGEL2* is likely to be a processed gene derived from *ModoMAGEL1*. Indeed, *ModoMAGEL1* and *ModoMAGEL2* form a monophyly in the tree (Fig. 1, Fig. S1) and in trees constructed by three different methods (NJ, RAxML and Bayes).

**Figure 2:**
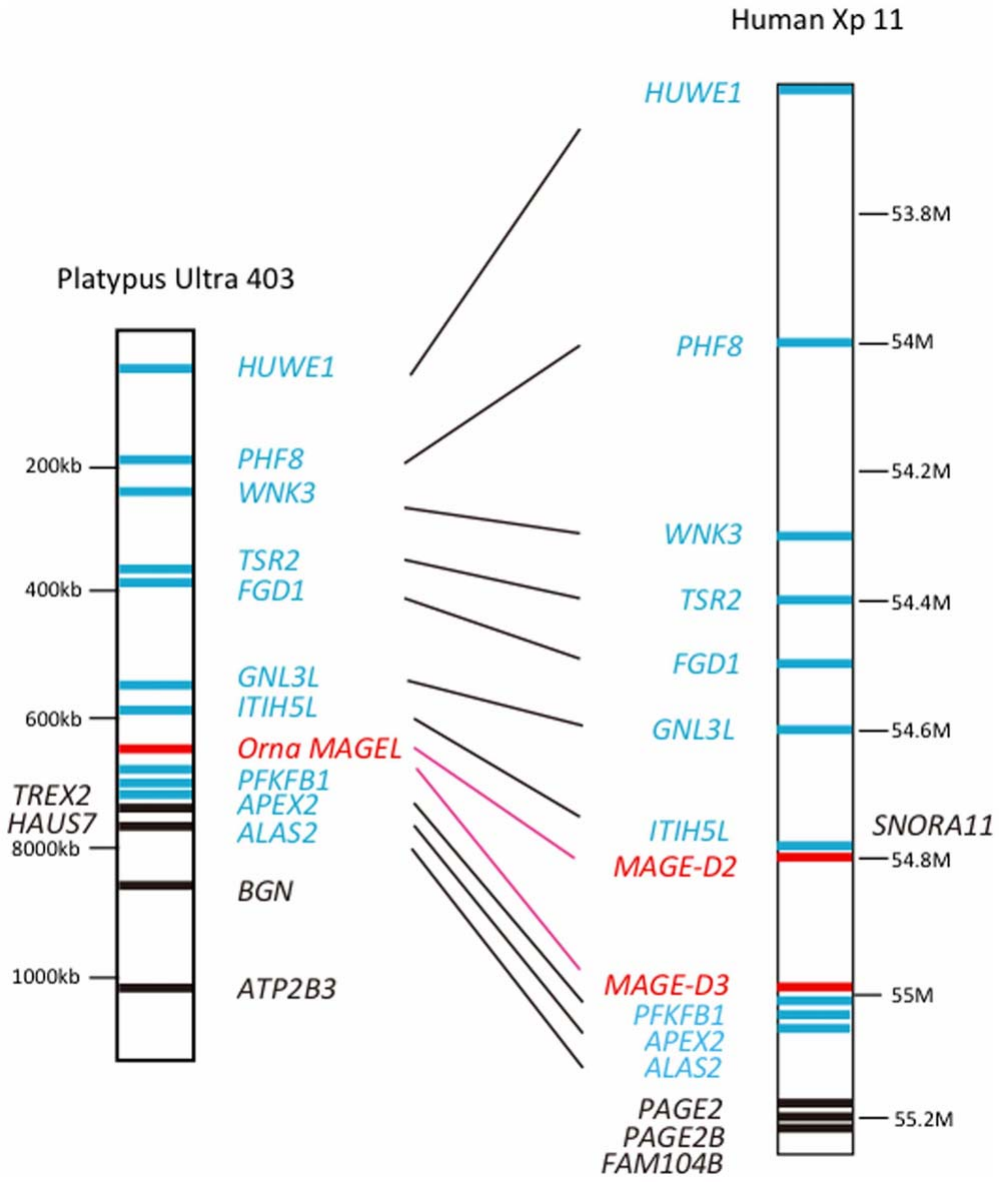
Synteny between platypus contig Ultra 430 and human X chromosome Xp11. Red bars indicate *MAGE-D* or *MAGEL* genes in the human or platypus, respectively. Black bars and gene names indicate syntenic genes between humans and platypuses. Blue bars and gene names indicate genes that do not show synteny. Other *MAGE-D* subfamily members, *MAGE-D1* and *MAGE-D4* are located at 51.6 M and 51.9 M on the human X chromosome, respectively.

The platypuses *OrnaMAGEL* gene is located on the contig Ultra 403 and consists of 10 exons. Although the number of exons differs from that in *ModoMAGEL1*, the phases and sizes of shared exons are well conserved (Table 1). Moreover, Ultra 403 also contains the ubiquitin ligase gene *HUWE1* (HECT, UBA and WWE domain containing 1), which is located ∼600 kb upstream from *OrnaMAGEL*. An *in situ* hybridization study confirmed that in the platypus, *HUWE1* is located on chromosome 6 [29]; thus, it is likely that this contig is a part of chromosome 6. Platypus chromosome 6 is homologous to the autosomal ancestor of eutherian and marsupial X chromosomes [29]. In fact, the region surrounding *OrnaMAGEL* on the contig showed a syntenic relationship with the human Xp11 region. In the human genome, the position corresponding to *OrnaMAGEL* is occupied by *MAGE-D2* and -*D3* (Fig. 2). Human *MAGE-D2* and -*D3* possess 13 exons, and the phases and sizes of shared exons are conserved with *OrnaMAGEL*, as well as with *ModoMAGEL1* and other *MAGE* genes in the chicken, frog, and zebrafish genomes (Table 1).

### Phylogeny of the mammalian *MAGE* gene family

**Figure 3:**
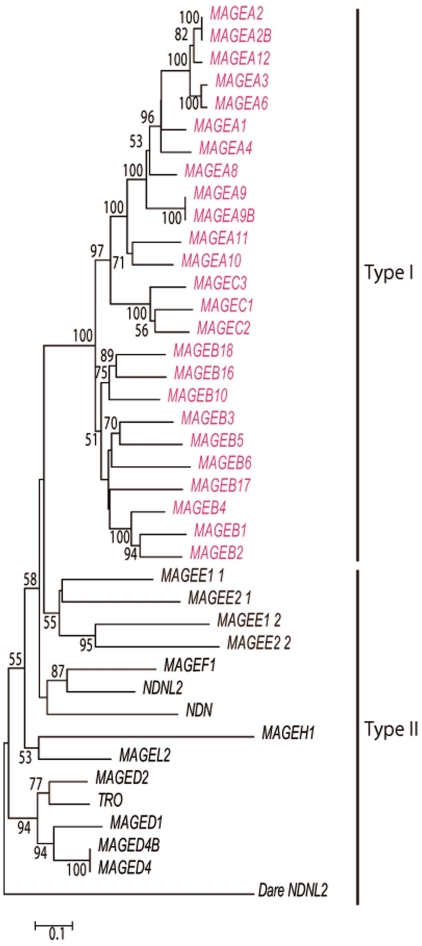
Phylogeny of MHD in human *MAGE* genes. The tree is based on the number of amino acid differences per site (*p*-distances). Genes but for *DareNDNL2* in the tree are all *MAGE* genes found in the human genome. *DareNDNL2* from zebrafish is used to determine the root of the tree. The number of sites compared is 92 amino acids without gaps. The bootstrap value is indicated at the node. Sequences are listed in Table S1. *MAGE-E* has duplicated MHD and the duplication has occurred earlier than the emergence of type Ι genes. *MAGEE1_1* (*MAGEE2_1*) and *MAGEE1_2* (*MAGEE2_2*) represent the MHD at the N and C terminal side of *MAGE-E1* (*MAGE-E2*), respectively. The eutherian *MAGE-D3* gene encodes trophinin (TRO), which is expressed in the placenta and affects embryo implantation.

A tree of human *MAGE* genes shows that the three type I *MAGE* subfamilies (*MAGE-A*, -*B* and –*C*) form a monophyletic cluster that is distinct from the seven type II subfamilies (*MAGE-D*, -*E*, -*F*, -*H*, -*L2*, *NDN* and *NDNL2*) (Fig. 3). The evidence is supported by five phylogenetically informative substitutions (D16Y, K23T, I62V, A113E, and R156Q in an alignment of the MHD, Fig. S3). In addition, *MAGE-D* genes form a monophyletic cluster. Although the number of nucleotides used in this analysis is small, it is clear that type I subfamilies diverged more recently than type II subfamilies (Fig. 3 and Fig. S2).

With the exception of *MAGE-D* genes, mammalian *MAGE* genes have a single exon for CDS. Thus these are likely to be processed genes derived from transcripts of *MAGE-D* or other *MAGE-D* processed genes [7], [30]. However, we cannot rule out the possibility that an ancestor of each subfamily resulted from duplication of a processed gene.

To examine how the ancestor of each gene family arose, the nucleotide sequences of a single representatives from each subfamily were compared with one another using dot-matrix analysis [16]. If an entire coding region including flanking region has been duplicated, the dotter analysis shows the similarity beyond the CDS. On the other hand, an ancestor of each subfamily has been generated by retrotransposition, the analysis shows the similarity only in the CDS.

For the most *MAGE* genes, the dot-matrix analysis revealed that within and between type I and II significant similarities were observed only in CDS regions, suggesting a retrotranspostion. A comparison between *MAGE-A* and *MAGE-C*, on the other hand, was an exception. The comparison reveals the similarity beyond the CDS, suggesting the DNA-based gene duplication. However, it might be possible that other subfamilies were also generated by gene duplication. The sequence similarity in flanking regions of duplicates was possibly lost during evolution because of the weaker functional constraint. Indeed, the extent of synonymous sequence divergences among type II gens and those between type I and type II genes ranges from 0.81 to 1.0, such that no significant similarity in a region beyond the CDS was observed. Although cladistic markers such as *LINEs* might have been informative for distinguishing retrotransposition from gene duplication, no such informative elements were found. Therefore, in the absence of any supportive evidences, we concluded that *MAGE-C* was duplicated from *MAGE-A* and that other subfamilies were generated by retrotransposition. In total, eight insertions of retrotransposed *MAGE* have occurred in the genome of ancestral Eutheria and each processed gene became an ancestor of a subfamily. Following retrotransposition, an independent gene duplication appears to have taken place within each subfamily.

### Gene duplication and palindrome formation

It is noteworthy that the clustering pattern of *MAGE-A* differs from that of *MAGE-B* (Fig. 1, Fig. S1). Each of the 11 human *MAGE-B* genes form a monophyletic cluster with orthologs in other eutherians, whereas the 15 *MAGE-A* genes form species- or taxon-specific clusters (Fig. 1, Fig. S1 and S2). Moreover, three *MAGE-C* genes appear to be primate-specific. Within the two type II *MAGE* subfamilies, five *MAGE-D* and two *MAGE*-*E* genes also show a clustering pattern (one-to-one orthologous relationship) similar to that of *MAGE-B* (Figs. 1 and 3).

**Figure 4:**
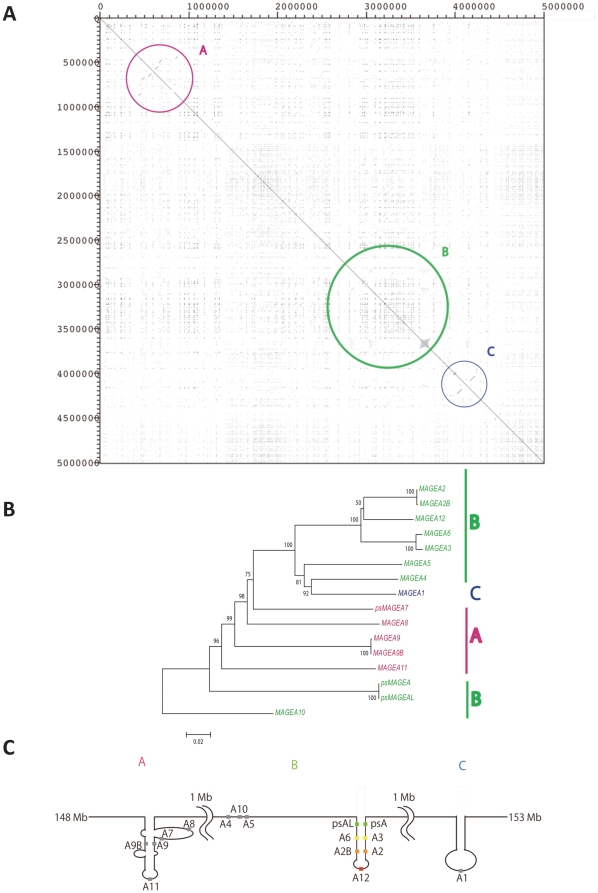
Genomic structure, palindrome prediction and phylogeny in human *MAGE-A* genomic region. (*A*) A diagonal line drawn from the upper left to the lower right indicates identity within the region. The region is divided into the three subregions, A, B, and C, which contain five, 10 and one *MAGE-A* genes, respectively. (*B*) The tree was constructed using the number of nucleotide differences (*p*-distances) among CDSs (1916 bp) of the 16 *MAGE-A* genes. The number at each node represents the bootstrap probability supporting that node. Bootstrap values greater than 50% are shown. Operational taxonomic units (OTU) in magenta, green and blue represent genes in subregions A, B and C, respectively. (*C*) Three predicted palindromes shown in subregions A, B and C. In subregion B, most of genes are located on putative palindrome arms.

A total of 16 *MAGE-A* genes are located on Xq28, in the region of 148 Mb to 153 Mb, and are clustered into three blocks A, B and C (Fig. 4A). Blocks A and B contain five (*MAGE*-*A11*, -*A9*, -*A9B*, -*A8* and *psMAGEA7*) and ten (*MAGE*-*A4*, -*A5*, -*A10*, -*A6*, -*A2B*, -*A2*, -*A12*, -*A3*, *psMAGEA* and *psMAGEAL*) genes, respectively, whereas block C contains a single gene (*MAGE-A1*) (Fig. 4B and C). Each of the three blocks possesses a palindrome (Fig. 4C). However, in only block B most genes (six out of ten) are located on both arms of the palindrome (Fig. 4C). Three nearly identical pairs of *MAGE*-*A2*/*A2B*, -*A3*/*A6*, *psMAGEA/psMAGEAL* are located in symmetric positions on the arms (Fig. 4B and 4C), whereas *MAGE-A12* is located in the loop region. We designated a pair of duplicate genes or sequences x and y on symmetric positions of the palindrome as x/y. The phylogenetic relationship among 16 *MAGE-A* genes including *psMAGEAL* (Fig. 4B, see Materials and Methods) and with *MAGE-D* used as an outgroup revealed that five genes in block B are in a monophyly, whereas a pair of *psMAGEA*/*psMAGEAL* genes are distantly related to other *MAGE-A* genes.

**Figure 5:**
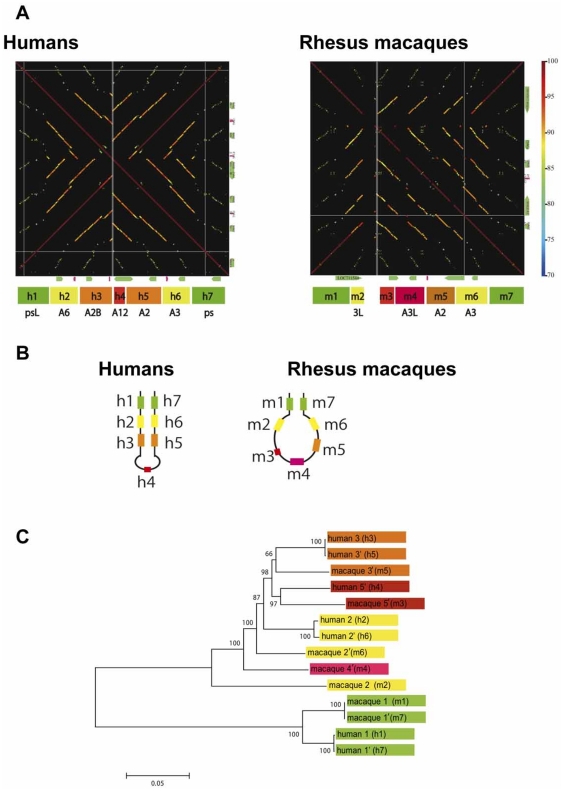
Genomic structures, phylogeny and predicted palindromes in subregion B. (*A*) The diagonal lines from the left top to the right bottom indicates identity within the human (left panel) or the macaque (right panel) sequence. Gaps in the diagonal line in the macaque indicate sequencing gaps. The colored boxes at the bottom of each panel indicate seven duplicated units. The same colored boxes within a species indicate that they are more closely related to each other than to others, whereas those between species indicate putative orthologs. (*B*) Palindromes predicted in subregion B of the human (left) or the macaque (right) sequence. Numbers beside the lines indicate each duplicated unit. (*C*) An NJ tree based on *p*-distances between duplicated units (2880 bp) is shown. The color-code for OTU is the same as in (*A*) and (*B*).

Human block B consists of seven duplicate units. Each unit is 10–20 kb long and contains a *MAGE-A* and a chondrosarcoma associated gene (*CSAGE*) [31] (Fig. 5*A*). BLAST analysis of mammalian genomes also shows the absence of *CSAGE* homologs in non-primate mammals. The palindrome in block B was not observed in non-primate genomes, such as the mouse, dog and horse genomes.

Among primates, block B can be identified in macaques (Fig. 5*A*). This block also contains seven duplicated units, but the form of the expected palindrome differs between the human and macaque. Unlike the long stem and short loop observed in the human, in the macaque, a short stem and a large loop structure is predicted (Fig. 5*B*). Further, the orthology of units between macaques and humans is curious given their positions. For convenience, we designated the seven duplicated units in block B as *h1* to *h7* in humans and *m1* to *m*7 in macaques (Fig. 5*A*) and then examined their phylogenetic relationships (Fig. 5*C*). Units of *h1*/*h7* harboring *psMAGEAL* and *psMAGEA* genes are orthologous to *m1*/*m7*. Units of *h3*/*h5* with *MAGE-A2/A2B* genes are orthologous to *m5* with *MAGE-A2*: however, in macaques, *m5* is located in the loop and there is no partner (a highly similar sequence) of *m5* in the block. The unit of *h4* with *MAGE-A12* is orthologous to *m3*, but in macaques this unit does not contain a *MAGE* gene (Fig. 5*A*). Furthermore, the relationships among *h2*/*h6*, *m*2, *m4* and *m*6 are somewhat confusing, despite the fact that the *MAEG-A3/A6* is in the *h2*/*h6* and three possible homologs (*MAGE-A3*, -*3L*, and –*A3L*) are in *m*2, *m4* and *m*6. The *p*-distance between *h2* and *h6* was 0.7% (±0.2), whereas the *p*-distances among *m*2, *m4* and *m*6 are much greater (12.1%) than the former. The pairwise distances of units between humans and macaques ranged from 8.3% (±0.5) to 17.7% (±0.7), which is too large for an orthologous relationship. The phylogeny also did not support an orthologous relationship among each of the three units in macaques (*m*2, *m4*, or *m*6) and *h2*/*h6* (Fig. 5*C*).

**Figure 6:**
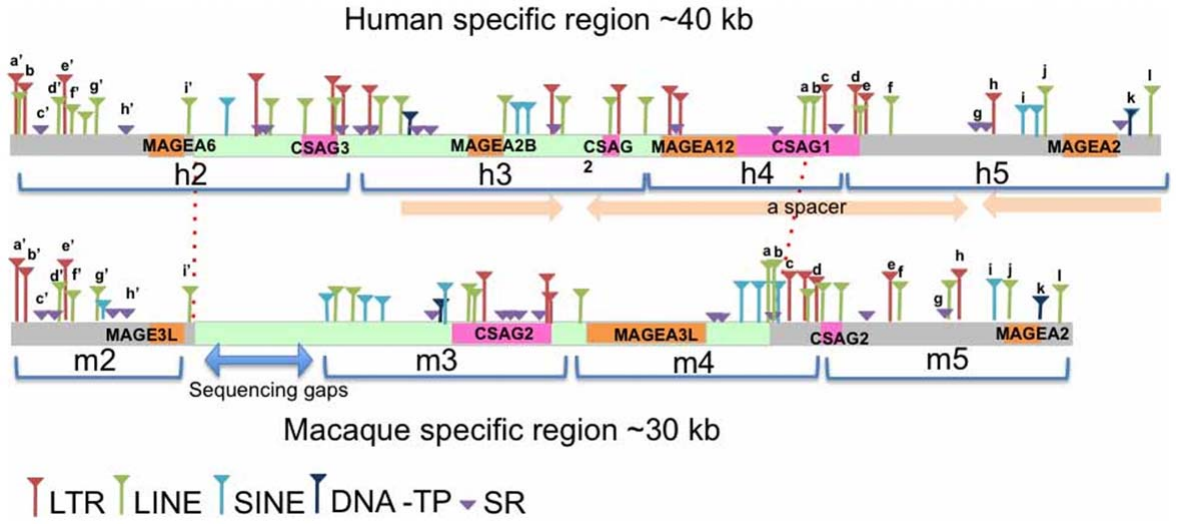
Maps of cladistic markers in humans and macaques. Colored triangles show interspersed elements (*LINEs* or *SINEs*), *LTRs*, DNA transposons (*DNA-TP*) or simple repeats (*SR*) found in the human or macaque genome, respectively. Brackets under each line indicate duplicated units. Light pink arrows indicate palindrome structure. The light blue arrow indicates sequencing gaps in macaques. Letters a to l and a′ to i′ on the triangles indicate orthologous insertion elements in the human and macaque genomes. The light green bar indicates a human- or macaque-specific region and dotted lines indicate the boundary between species-specific and orthologous regions.

To further examine the orthologous relationships of these duplicated units, cladistic markers such as *SINE*s and *LINE*s were sought using RepeatMasker software [24] (Fig. 6). In general, the arrangement of *SINE*s, *LINE*s, *LTR*s, and short repeats in block B shows partial similarity between the human and macaque genome. The position and type of repetitive sequences found across the entire *m2* region are almost identical to those found in the distal half of *h2*. A similar distribution of repetitive sequences is observed between a region of *m5* and *h5*, and the similarity is also observed between a part of *m4* and that of *h4*. However, species-specific regions seem to be present in each genome. In humans, the region is ∼40 kb long and extends from the middle of *h2* to *h4*, while in macaques, the species-specific region is ∼30 kb and extends from the middle of *m2* to *m4*. Unlike results of the phylogeny and genetic distance analyses (Figs. 5*C* and 6), the cladistic markers showed that *h2* with human *MAGE-A6* and *m2* with the macaque *MAGE3L* are indeed orthologous to each other.

### Human-specific palindrome and gene conversion

**Figure 7:**
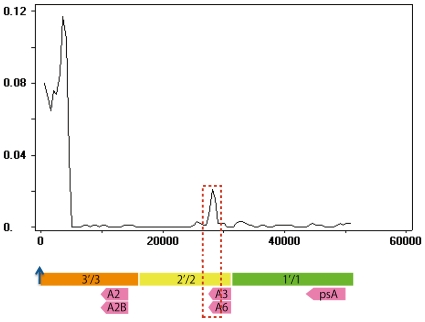
Window analysis of nucleotide divergence between a pair of palindrome arms in the human genome. The window size is 500 bp with no overlap between adjacent windows. Colored rectangles at the bottom of the figure indicate the duplicated unit including the *MAGE* genes (light pink arrows). The ordinate represents nucleotide divergence (*d*) and the abscissa represents position (in bp) relative to the center of the loop (position zero, blue arrow). The area surrounding a red dotted line indicate the high diverged region in *MAGE-A3* and *MAGE-A6*.

The dot-matrix analysis revealed that the palindrome in block B is apparent only in humans. Although sequencing gaps currently exist in the chimpanzee and orangutan genome, the available sequences showed that the palindrome in block B is less apparent in these two apes than in humans (Fig. 7). We parsimoniously inferred the ancestral state of the palindrome by using sequence information of the genome of extant primate species.

**Figure 8:**
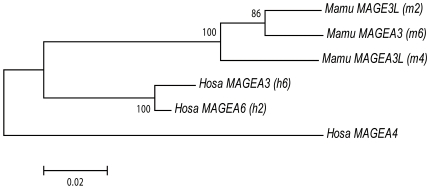
The phylogeny of six *MAGE-A* genes from humans and macaques. The NJ tree was based on synonymous divergences among six *MAGE-A* CDSs. The number of sites compared is 314. The root is determined by *MAGE-A4*.

Genes on palindromes may experience frequent gene conversion. Indeed, a window analysis of 500 bp with a non-overlapping interval reveals that sequences of palindrome in arms are almost identical (Fig. 8). Furthermore, analysis with a program GENECONV also revealed a possible gene conversion in the majority of palindrome arms. However, in the middle of *h2* and *h6*, there is a region with significantly large sequence divergence (p = ∼2%, P<0.001) compared with the neighborhood (Fig. 8). The highly diverged region corresponds to a 673 bp of the 5′ ends of the *MAGE-A3*/*A6* sequences. *MAGE-A3*/*A6* encode epitopes for HLA class I molecules in tumor cells and for epitopes for HLA class II molecules in melanoma cells [32]–[34]. The distribution of these epitopes in type I *MAGE* genes (Fig. S4) reveals that epitope-coding is confined to this highly diverged region (Fig. S4). In fact, among 13 amino acid changes between MAGE-A3 and -A6, 10 substitutions are concentrated in this epitope-coding region. Both MAGE-A3 and -A6 can thus produce various epitopes for many kinds of HLA molecules (Fig. S4).

## Discussion

### The ancient origin of *MAGE* genes (phase I)

The search for a *MAGE* gene in invertebrate genomes has revealed the presence of homologs in the tunicate and the lancelet genomes. *MAGE*-like genes containing the conserved MHD sequence have also been reported in insects [9], [10]. The *MAGE* gene in the fruit fly (*Drosophila melanogaster*: *DrmeMAGE*) plays a key role in neurogenesis [35]. The gene lacks an intron and therefore might be a processed gene. We searched for a *DrmeMAGE*-related gene with introns in the FlyBase data (http://flybase.org/), but no candidate gene was detected. We also carried out a TBLASTN search for a *DrmeMAGE* homolog with introns over the entire NCBI database. We found that the MHD in *DrmeMAGE* has nearly 30% similarity with MHD in vertebrate *MAGE* genes, and that the amino acid sequence of the epitope-coding region in the human *MAGE-B16* (FLWGPRAKAE) [10] is completely conserved. However, *DrmeMAGE* is not expressed in tumor cells and it does not code for antigens in the fly. *MAGE* homologs were also found in the *Arabidopsis thaliana* genome. *A. thaliana MAGE* shares 25% similarity with human *MAGE-A8* but the function of the *MAGE-*related gene is not known. Since our study showed that the *MAGE-A* and -*B* subfamilies diverged in eutherians, *MAGE*-like sequences in insects and plants have originated independently from eutherian *MAGE*-*A* and -*B* sequences and the extent of sequence similarity between *MAGE* genes in eutherians and in plants or insects might be due to functional convergence.

The synteny (Fig. 2) and the conservation of phases in exons (Table 1) reveal that *OrnaMAGEL* is an ortholog of *MAGE-D2* or -*D3* in humans. An ancestral *MAGE* gene probably had been a single copy until the divergence of monotremes and therians. Moreover, in the stem lineage of mammals, the ancestral *MAGE* was located on an autosome that later differentiated into a sex chromosome. Thus, the *MAGE* gene presumably become X-linked in marsupials and eutherians, and differentiated into *MAGE-D* in extant eutherians. The eutherian *MAGE-D3* gene encoding trophinin (TRO) is expressed in the placenta and affects embryo implantation [36], suggesting that *MAGE-D3* evolved its current function specifically in eutherians.

Since the ancestral *MAGE* gene was on the proto-X chromosome, the gene may have a homolog on the extant Y chromosome. This is because both sex chromosomes are thought to have derived from a pair of autosomes. However, we found no *MAGE* homolog on the Y chromosome of humans or other eutherians. The region syntenic to human Xp11 is located near the tip of the opossum X chromosome. However, in many eutherians the regions syntenic to human Xp11 are located near the centromere of the X chromosome. The ancestral region appears to have moved towards the centromere before the radiation of eutherians. This transposition on the X chromosome may have prevented pairing with the Y chromosome, leading to loss of *MAGE* from the Y chromosome.

### Formation of ancestors of multi-gene families by retrotransposition (phase II)

In eutherians, the *MAGE* gene family can be divided into 10 subfamilies. Nine of these subfamilies, all but *MAGE-D*, are processed genes and then ancestors of eight subfamilies, all but *MAGE-D* and *MAGE-C*, appear to have been generated via retrotransposition. The source of the retrotransposed genes has been thought to be *MAGE-D* [7]. We attempted to conform both the source of these genes and their order of emergence using the extent of similarity among the CDSs of *MAGE* genes. However, the stretches of sequences with significant similarity were too short to make conclusion about the ancestry of processed genes.

At least eight times of retrotransposition may have been necessary to produce ancestors of each of the eight extant *MAGE* subfamilies at the early stage of eutherian evolution. The activation of reverse transcriptase necessary for this transposition might have been provided by the activation of *LINE* elements at that time [37].

To be functional, any processed gene should gain promoter activity near the insertion site. *MAGE-A*, -*B*, and -*C* are all expressed in cancer cells and in the testis. Sequence similarity beyond the CDS shows that *MAGE-A* and *MAGE-C* were produced by gene duplication. In addition, the tumor types in which *MAGE-A* is expressed are similar to those in which *MAGE-C* is expressed, but different from those in which *MAGE-B* genes are expressed [15], [38], [39]. Based on the similar pattern between *MAGE-A* and *MAGE-C* gene expression, conserved TFB sequences are expected in the upstream region of *MAGE-A* and –*C*. Indeed, in the ∼400 bp upstream of ATG, *MAGE-A* and *-C* have potential TFBs in common. Among several such TFBs, STAT (signal transducers and activators of transcription) binding site (TTCCCRKAA) and LYF (lymphoid transcription factor) binding site (TTTGGGAGR) are found. These sequences are known to act in cancer cells [40], [41].

### Gene duplication and palindrome formation (phase III)

The high sequence similarity over the flanking region including possible regulatory elements and the monophyly of *MAGE-A* genes in the phylogeny (Figs. 1 and 3) suggest that *MAGE-A* subfamily members most likely originated from gene duplication. Nucleotide divergences among *MAGE-A* genes (10 to 15%) show that most *MAGE-A* genes emerged in the stem lineage of Catarrhini or even earlier. Thus we reasoned that orthologs of *MAGE-A* genes might be present in New World monkeys as well. A database search for such homologs revealed three sequences on contigs 7129, 6382 and 5036 in the common marmoset genome (*Callithrix jacchus*, *UCSC* WUSTL version Callithrix jacchus-2.0.2) with greater than 80% similarity to *MAGE-A2*/*A2B*, *A3*/*A6* and *-A12*. Moreover, three additional sequences on contig 880 and one sequence on contigs 1178 and 6382 also show 76–79% similarity to several human *MAGE* genes. Thus, a total of eight *MAGE-A* homologs were detected in the common marmoset genome. Although the genomic locations of these homologs are not yet known, their presence is consistent with the idea that the duplication that produced a set of *MAGE*-*A* genes probably took place in the stem lineage of simian primates.

It is worth noting that large palindromes on the Y chromosome have also been generated in the stem lineage of the Catarrhini or even earlier [42]. The eight palindromes on the human Y chromosome contain seven gene families. Although nucleotide sequences in symmetrical positions on the palindromic arms are nearly identical, gene family members in asymmetric positions show nucleotide divergences ranging from 5.9 (±1.0) to 13.9% (±1.5). This range is similar to those observed between duplicated units in humans or macaques on the X chromosome, suggesting that simultaneous gene duplication on the X and Y chromosome may have occurred.

The phylogeny of the CDSs of human *MAGE*-*A3*/*A6* and macaque *MAGE*-*A3*, -*3L*, and -*A3L* genes indicates that they diverged in the stem lineage of the Catarrhini (Fig. 8), yet their synonymous nucleotide differences are exceptionally high (*p* = 13.4% (±2.2), Table S2). As is often observed in newly duplicated genes, the degree of functional constraint may change and permit frequent substitutions in CpG dinucleotides. This appears to have happened in the present case as well. Among 315 codons in these *MAGE* genes, 45 codons contain CpG sites. If the latter codons are excluded, synonymous divergence decreases between human *MAGE*-*A3* or -*A6* and macaque *MAGE*-*A3*, -*3L* or -*A3L* to 7.8% (±2.1) (Table S2, ranging from 6.4% (±1.8) to 9.3% (±2.4)), which is not significantly different from the overall average divergence between human and macaque X chromosomal genes (5.5% (±0.3)) [43]. These results confirm orthology among human *MAGE*-*A3*/-*A6* and macaque -*A3*, -*3L*, and -*A3L* genes. Importantly, the analysis of syntenic *LINE* and *SINE* insertions also clearly indicates one-to-one orthology between *MAGE-3L* in macaques and *MAGE-A6* in humans (Fig. 6).

### Human specificity in a palindrome (phase IV)

**Figure 9:**
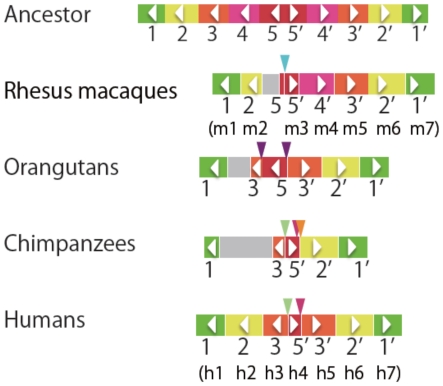
Inferred rearrangements in primate *MAGE-A* genomic subregion B. A schematic diagram of duplicated units containing *MAGE-A* genes in an ancestral and extant species is shown. Each colored box indicates a different duplicated unit as in Fig. 5*A*. Gray bars indicate sequencing gaps. Colored triangles indicate independent deletions. The same colored triangle in chimpanzees and humans indicates that the deletion occurred in an ancestral species. An arrowhead in each rectangle indicatte the direction of the fragment.

The overall sequence divergences among orthologous duplicated units in humans and macaques exceed 10%. Since both humans and macaques have seven units of duplicates, it is assumed that five pairs of duplicated units already formed a palindrome in the ancestral genome (Fig. 9). Under this assumption, the present arrangement of duplicated units suggests species- or lineage-specific deletions in a loop region of the palindrome (Fig. 9).

Further examination of nucleotide divergence between the palindromic arms in humans reveals the presence of a significantly diverged region in the middle of *MAGE-A3* and *-A6* (Fig. 7). Four synonymous substitutions have accumulated exclusively at CpG sites between *MAGE-A3* and *-A6*, and 22 synonymous substitutions differentiate the human *MAGE-A6* from the macaque *MAGE-3L*. If these 22 substitutions have accumulated over the period of 35 million years (myr) of divergence between the two species [44]–[46], then the accumulation of four substitutions corresponds to 6.4 myr (35 myr×4/22). This suggests that the divergence between *MAGE*-*A3* and -*A6* in humans occurred when humans diverged from chimpanzees (∼6 to 7 MYA) [47]. Although a one-to-one ortholog of human *MAGE*-*A6* have not been identified in the chimpanzee genome, chimpanzee *MAGE3* (a one-to-one ortholog to human *MAGE-A3*) apparently encodes a lower variety of epitopes than the human ortholog (Fig. S4). In chimpanzees, *MAGE3* is indeed truncated in is CDS. Thus, it is likely that the nucleotide differences between *MAGE-A3* and *-A6* have accumulated specifically in humans.

These findings lead to questions about the evolutionary forces maintaining the diversity observed in human *MAGE*-*A3* and -*A6*. Considering the role of MAGE-A proteins in cancer immunity [4], a diversity of epitopes might be advantageous. *MAGE-A* must encode variable epitopes to maintain their ability to bind to HLA molecules. Two alternative mechanisms for generating diversity can be considered: Darwinian selection elevating nonsynonymous substitutions or negative selection against homogenization by gene conversion. Darwinian selection might operate on this epitope-encoding region to enhance the accumulation of mutations.

To know whether Darwinian or negative selection operates we examined the relative rates of nucleotide substitutions in *MAGE-A3* and -*A6* and their flanking region using the *MAGE-A2* sequence as a reference. If Darwinian selection operates in the epitope-coding region, then nucleotide divergence in the epitope-coding region with being compared to *A*2 should be higher than in the remaining non epitope-coding region. However, we found that the substitution rate is not higher at nonsynonymous sites in the epitope-coding region between -*A2* vs. -*A3* and -*A2* vs. -*A6* than at those in the non epitope-coding region. The same result was obtained using different *MAGE-A* genes as references. Thus, we conclude that the divergence between *MAGE-A3* and -*A6* was not generated by an elevated nonsynonymous substitution rate. This is also supported by the ratio of nonsynonymous to synonymous divergences between *MAGE-A3* and -*A6* (*d_N_/d_S_* = 0.9, P<0.001, H_0_: *d*_N_ = *d*_S_). Rather highly diverged epitopes between *-A3* and *-A6* indicate negative (purifying) selection against homogenization by gene conversion. A similar effect of negative selection has been observed in immunoglobulin genes [2].

### Co-evolution between HLA and MAGE epitopes

Evoking negative selection strongly argues for co-evolution between *HLA* and *MAGE-A3* or -*A6*. A variety of epitopes must be present in each a MAGE protein, because *HLA* is extraordinary polymorphic and the *HLA* and *MAGE* genes are located on different chromosomes; They are in unlinked status. Because of this unlinked status, it would be difficult for MAGE to be polymorphic in order to associate with *HLA*.

*MAGE-A3* and -*A6* encode seven different kinds of epitopes to bind seven different HLA class I molecules: HLA-A1, -A24, -A2, -B37, -B52, -B44, and -B35 molecule. Curiously, however, in macaques there are no corresponding allelic lineages producing the seven major histocompatibility complex (*MHC*: *HLA* homologs in macaques) molecules (data not shown). Thus, the association between *MAGE* and *MHC* in macaques might be different from those observed in humans. This evolutionary mode of primate *MAGE-A* genes may be associated with rapid turnover of *HLA* class I loci in the primates [48]. In addition, among epitope-coding *MAGE*s, *MAGE-A3* and *-A6* are unique in that they are highly expressed in tumor cells and encode the highest number of identified epitopes in a gene [32], This might explain reasons why negative selection against gene conversion appears to have operated on only *MAGE-A3* and *-A6*.

The human-specific genetic diversification between *MAGE-A3* and -*A6* on the palindrome may be associated with human evolution. After diverging from chimpanzees, human ancestors were still arboreal. Subsequently, these ancestors left the forests to live in the savanna and later they lost their fur. This change in habitat likely resulted in direct exposure of the naked skin to strong ultra-violet light. Such exposure is known to increase the risk of tumors such as melanoma. As a means of protection against tumor progression, it is reasonable to imagine that various MAGE-A3 and -A6 genes would be favored by natural selection, facilitating HLA-mediated immunity.

### Unique mode of evolution in the *MAGE* gene family

In the human genome, there are many gene families that appear to have been generated by gene duplication and retrotransposition. Well-known examples of the former case include a set of genes for ribosomal RNAs [49], [50], and those for alpha and beta hemoglobins [51], [52]. In the case of ribosomal RNAs, the requirement for a large amount of the gene products causes the multiplication and homogenization of duplicated units. On the other hand, sequence divergence among members in the hemoglobin gene family depends on the requirement for physiological differentiation of proteins. This kind of functional diversification in a multi-gene family is quite common.

As discussed here, the multiplication of *MAGE* genes appears to have been mediated by both retrotransposition and gene duplication. Some members of the family have been homogenized by gene conversion, whereas others have evolved against it. The evolutionary mode appears to be determined by genomic environments such as palindrome formation, as well as by functional differentiation, such as to generate a variety of epitopes related to cancer immunity.

## Supporting Information

Figure S1 Enlarged version of Figure 1.
(TIF)

Figure S2 Schematic representation of the *MAGE* gene family diversification history. Each triangle indicates a subtree of the depicted subfamily. Numbers at the branch nodes indicate bootstrap values. Branch lengths are arbitrary and do not reflect evolutionary distances.
(TIF)

Figure S3 The MHD amino acid sequence alignment in human *MAGE* genes. A dot (.) indicates that an amino acid residue is the same as that in the top line. A dash (-) indicates a deletion of the residue at that position. Red characters indicate amino acid substitutions supporting a monophyletic relationship of *MAGE-A*, -*B* and -*C* (see text) [53]–[68].
(PDF)

Figure S4 An alignment of primate MAGE-A amino acid sequences for an epitope coding region. In humans, based on references (1–16), MAGE-A epitopes for HLA alleles are denoted by squares (magenta; HLC class Ι, light blue; HLA class II). HLA alleles that recognize each epitope are indicated in parallel below. Among 13 amino acid substitutions between *MAGE-A3* and -*A6*, 11 substitutions marked by stars occur in the alignment whereas two substitutions (P303L, A308V) occured outside of the region. Among the 11 substitutions, ten that contribute to the production of epitopes for different HLA alleles (E115K, D156L, L175V, T199A, L201F, V205I, K211R, D249H/D249Y, L279V/L279I, H298R) are indicated by green stars. The other substitution within this region (indicated by a blue star; F239L) does not contribute to the production of epitopes of MAGE-A3 and -A6 [53–68].
(TIFF)

Table S1 Accession numbers of nulceotide sequences used in this study. Species names are shown in colored cells.
(PDF)

Table S2 Nucleotide divergence among six *MAGE-A* genes from humans and macaques. Synonymous nucleotide divergences (below diagonal) and synonymous nucleotide divergences with removal of CG codons (upper diagonal) for the six *MAGE-A* genes were showed. Standard errors are provided in parentheses. Sequences are from humans (Hosa) and macaques (Mamu). The number of synonymous sites with CG codons is 226 and that without CG codons is 173.
(PDF)
