# Supplementary figures and images for: Evolutionary History of the Cancer Immunity Antigen MAGE Gene Family
Source: PLoS One. 2011 Jun 10;6(6):e20365. doi: 10.1371/journal.pone.0020365 (PMC3112145; doi:10.1371/journal.pone.0020365)

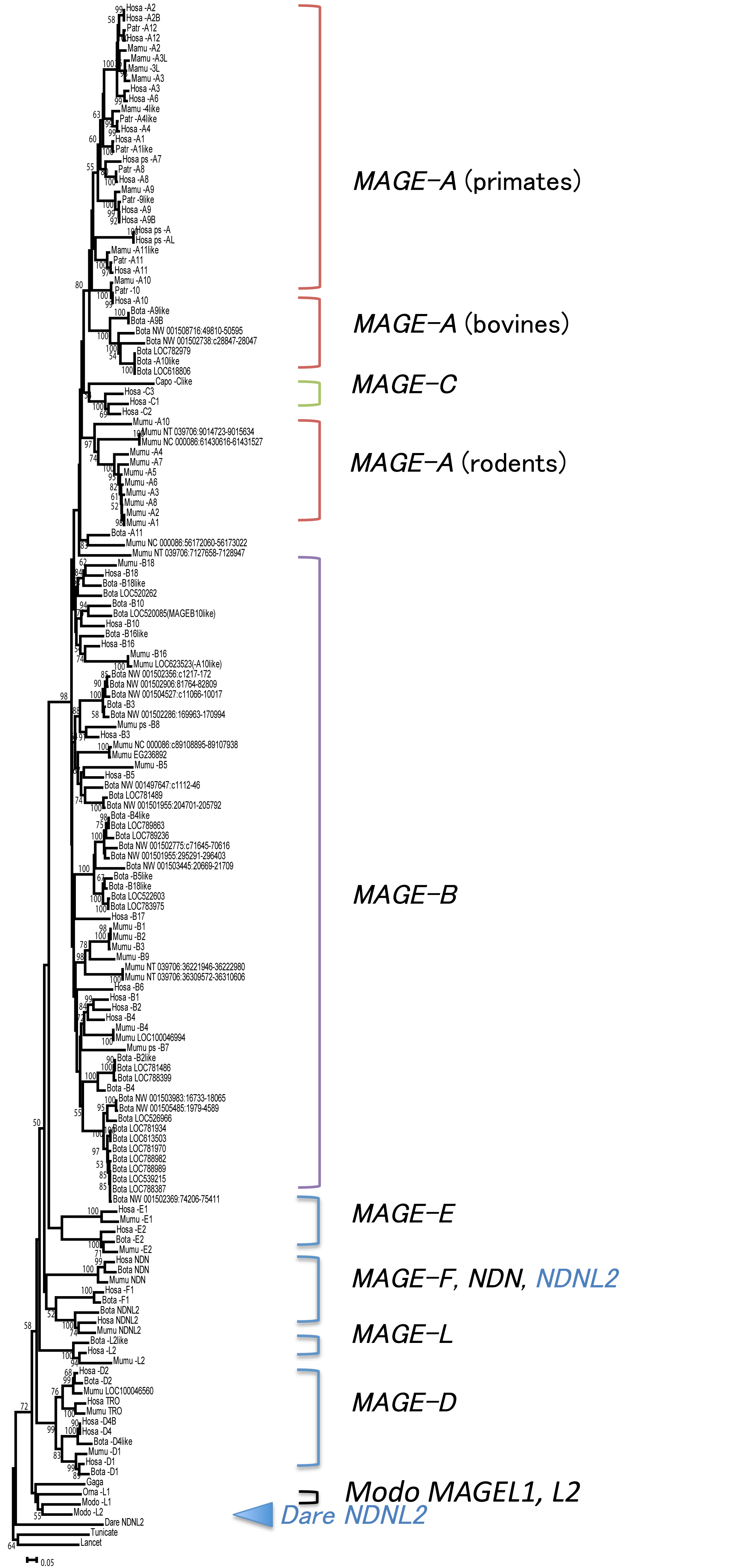

Supplement: Figure S1 — Enlarged version of Figure 1. (TIF) [file pone.0020365.s001.tif]

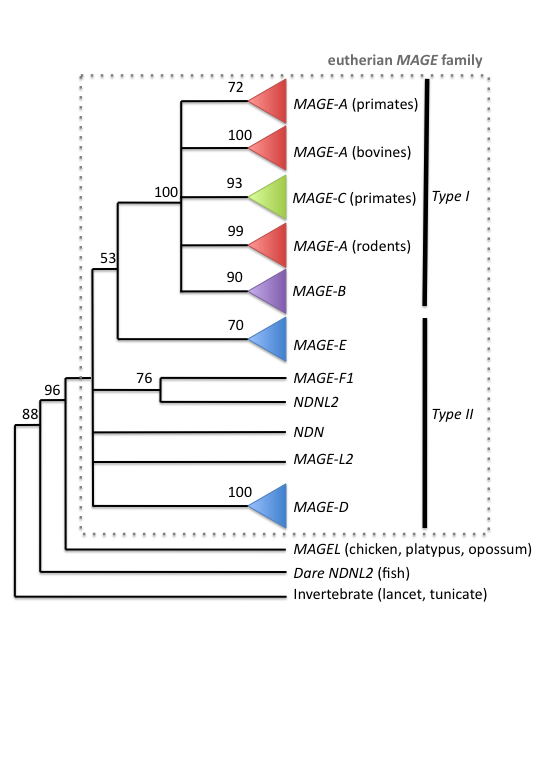

Supplement: Figure S2 — Schematic representation of the MAGE gene family diversification history. Each triangle indicates a subtree of the depicted subfamily. Numbers at the branch nodes indicate bootstrap values. Branch lengths are arbitrary and do not reflect evolutionary distances. (TIF) [file pone.0020365.s002.tif]

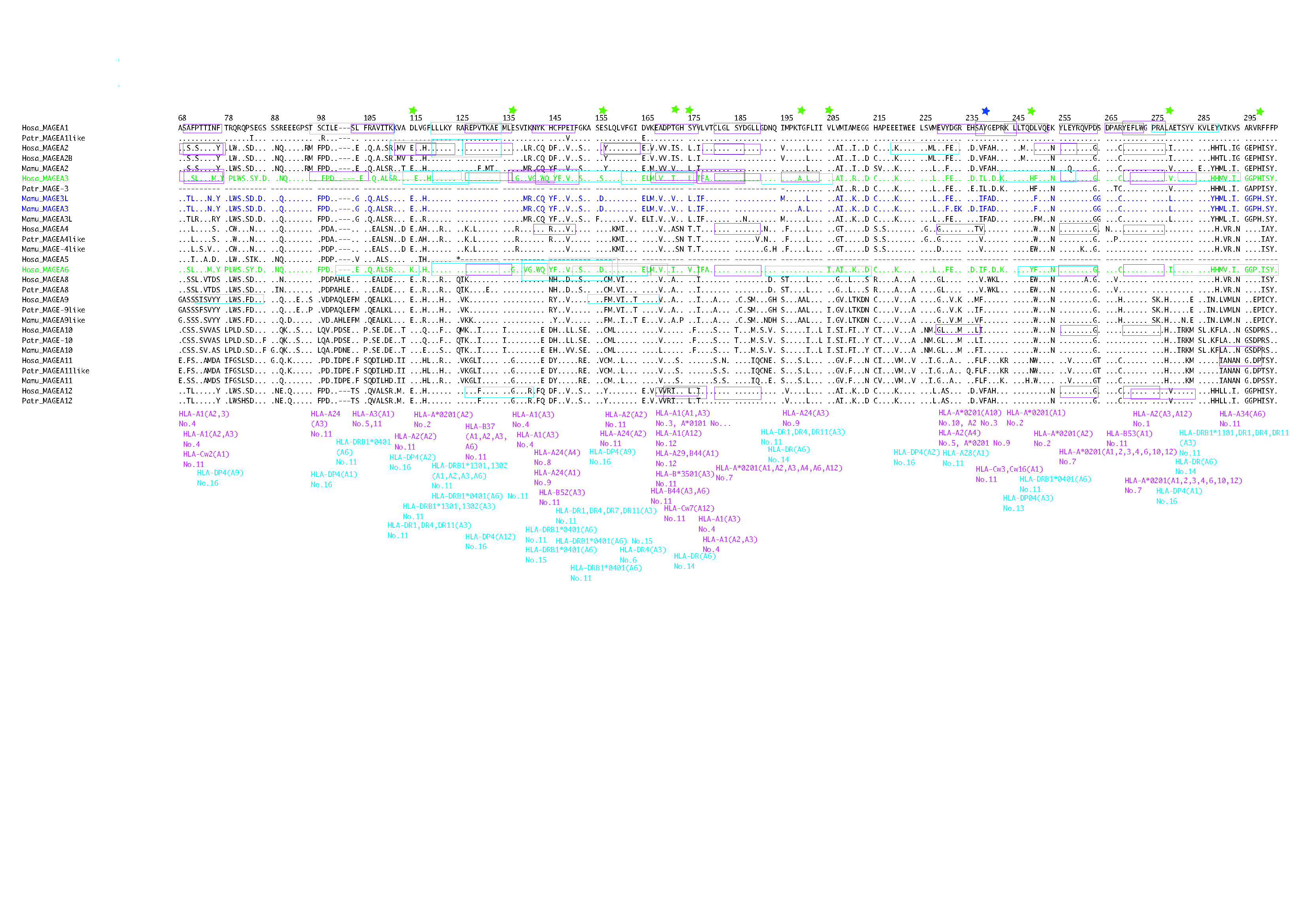

Supplement: Figure S4 — An alignment of primate MAGE-A amino acid sequences for an epitope coding region. In humans, based on references (1–16), MAGE-A epitopes for HLA alleles are denoted by squares (magenta; HLC class Ι, light blue; HLA class II). HLA alleles that recognize each epitope are indicated in parallel below. Among 13 amino acid substitutions between MAGE-A3 and -A6, 11 substitutions marked by stars occur in the alignment whereas two substitutions (P303L, A308V) occured outside of the region. Among the 11 substitutions, ten that contribute to the production of epitopes for different HLA alleles (E115K, D156L, L175V, T199A, L201F, V205I, K211R, D249H/D249Y, L279V/L279I, H298R) are indicated by green stars. The other substitution within this region (indicated by a blue star; F239L) does not contribute to the production of epitopes of MAGE-A3 and -A6 [53–68]. (TIFF) [file pone.0020365.s004.tif]
