# Supplementary material for: Evolutionary History of the Cancer Immunity Antigen MAGE Gene Family
Source: PLoS One. 2011 Jun 10;6(6):e20365. doi: 10.1371/journal.pone.0020365 (PMC3112145; doi:10.1371/journal.pone.0020365)
Supplement: Figure S3 — The MHD amino acid sequence alignment in human MAGE genes. A dot (.) indicates that an amino acid residue is the same as that in the top line. A dash (-) indicates a deletion of the residue at that position. Red characters indicate amino acid substitutions supporting a monophyletic relationship of MAGE-A, -B and -C (see text) [53]–[68]. (PDF) [file pone.0020365.s003.pdf]

```
                 1          11         21         31         41         51         61         71         81         91         101
Hosa_MAGEC1      LDEKVDELAR FLLLKYQVKQ PITKAEMLTN VI-SRYTGYF PVIFRKAREF IEILFGISLR EVDPD-DSYV FVNTLDLTSE GCLSDEQGM- SQNRLLILIL SIIFIKGTYA
Hosa_MAGEC2      .....A..VE ......EAEE .V.E....MI ..-K-.KD.. ...LKR.... M.L...LA.I ..G..HFC-. .A..VG..D. .--..DE..- PE.S...I.. .V.....NC.
Hosa_MAGEC3      .....A..VQ .......T.E .V.......T ..-KK.KD.. .M..G..H.. ..LI...A.T DM...NH..F .ED.....Y. .S.I.D...- PK.C...... .M.....SCV
Hosa_MAGEA1      ITK..AD.VG ......RARE .V......ES ..-KN.KHC. .E..G..S.S LQLV...DVK .A..TGH... L.TC.G.SYD .L.G.N.I.- PKTGF..IV. VM.AME.GH.
Hosa_MAGEA2      ISR.MV..VH ......RARE .V......ES .L-RNCQDF. ....S..S.Y LQLV...EVV ..V.ISHL.I L.TC.G.SYD .L.G.N.V.- PKTG...IV. A..A.E.DC.
Hosa_MAGEA3      .SR..A..VH ......RARE .V......GS .V-GNWQYF. ....S..SSS LQLV...E.M ....IGHL.I .ATC.G.SYD .L.G.N.I.- PKAG...IV. A..ARE.DC.
Hosa_MAGEA2B     ISR.MV..VH ......RARE .V......ES .L-RNCQDF. ....S..S.Y LQLV...EVV ..V.ISHL.I L.TC.G.SYD .L.G.N.V.- PKTG...IV. A..A.E.DC.
Hosa_MAGEA6      .SR..AK.VH ......RARE .V......GS .V-GNWQYF. ....S..SDS LQLV...E.M ....IGHV.I .ATC.G.SYD .L.G.N.I.- PKTGF..I.. A..AKE.DC.
Hosa_MAGEA12     .SR.MA..VH ......RARE .F......GS ..-RNFQDF. ....S..S.Y LQLV...EVV ..VRIGHL.I L.TC.G.SYD .L.G.N.IV- PKTG...IV. A..AKE.DC.
Hosa_MAGEA8      .....A..V. ...R..I.E .V......ES ..-KN.KNH. .D..S..S.C MQVI...DVK ....AGH..I L.TC.G.SYD .L.G.D.ST- PKTG...IV. GM.LME.SR.
Hosa_MAGEA4      .SN......H ...R..RA.E LV......ER ..-KN.KRC. ....G..S.S LKMI...DVK ....ASNT.T L.TC.G.SYD .L.GNN.IF- PKTG...IV. GT.AME.DS.
Hosa_MAGEA9      .KL..A..VH ...H..R..E .V......ES ..-KN.KR.. ....G..S.. MQVI..TDVK ....AGH..I L.TA.G.SCD SM.G.GHS.- PKAA...IV. GV.LT.DNC.
Hosa_MAGEA10     I....TD.VQ ...F...M.E ......I.ES ..-KN.EDH. .LL.SE.S.C MLLV...DVK ....TGH.F. L.TS.G..YD .M...V.S.- PKTGI..... .....E.YCT
Hosa_MAGEA11     .HD.IID.VH L..R..R..G L.......GS ..-KN.ED.. .E...E.SVC MQL....DVK ....TSH... L.TS.N.SYD .IQCN..S.- PKSG...IV. GV..ME.NCI
Hosa_MAGEA9B     .KL..A..VH ...H..R..E .V......ES ..-KN.KR.. ....G..S.. MQVI..TDVK ....AGH..I L.TA.G.SCD SM.G.GHS.- PKAA...IV. GV.LT.DNC.
Hosa_MAGEB1      VAWEAGM.MH .I.R..KMRE ..M..D..KV .D-EK.KDH. TE.LNG.SRR L.LV..LD.K .DN.SGHT.T L.SK.N..ND .N..NDWDF- PR.G..MPL. GV..L..NS.
Hosa_MAGEB4      .TR.TKM.VQ ...Y..KM.E .T......KI IS-KK.KEH. .E....VSQR T.LV..LA.K ..N.TTH..I L.SM.G-PND .NQ.SAWTL- PR.G..MPL. .V..LN.NC.
Hosa_MAGEB3      .IM.TNM.VQ ..MEM.KM.K ..M..D..KI .Q-KSHKNC. .E.LK..SFN M.VV..VD.K K..STK.... L.SKM..PNN .TVTRGR.F- PKTG..LNL. GV..M..NC.
Hosa_MAGEB2      .TR.SGS.VQ ...Y..KI.K SV..G...KI .G-K.FREH. .E.LK..S.G LSVV..LE.N K.N.NGHT.T .IDKV...D. ES.LSSWDF- PRRK..MPL. GV..LN.NS.
Hosa_MAGEB6      VKK.ACT..Q ..QK.FEK.E S.L..D..KC .R-RE.KP.. .Q.LNRTSQH LVVA..VE.K .M.SSGE..T L.SK.G.P.. .I..GDNAL- PKSG..MSL. VV..MN.NC.
Hosa_MAGEB18     .NK..VS.VH ...Q..ET.E ....GD.IKF ..-RKDKCH. NE.LKR.S.H M.LAL.VD.K ....IRHY.A .FSK....YD ETT...EKI- PKTG..MIA. GV..LN.NR.
Hosa_MAGEB16     ..Q..AF.VN .M.H.C.M.K .....D..KI I.-KDDESH. SE.LLR.S.H L.MI..LDVV ....TTHC.G LFIK.G..YD .M..G.K.V- PKTG...IV. GV..M..NR.
Hosa_MAGEB10     -....II.VH Y..Y...M.E .....D..R. .T-QMSKSQ. ...LSR.S.H L.LI..LD.K ..E.NKHI.. L..K...GCD AK....T.V- PKTG..MTV. G...TN.NCV
Hosa_MAGEB17     .NT.TG..VQ ...N..IR.E ...REA..KV IN-RK.KQH. .E.L.RST.N V.VV..LY.K .M..SRQ... L.GK..FPNQ .S...GG.F- PLSG..MVL. .T..MH.NR.
Hosa_MAGEB5      INI..GL.EQ ...Y.FKM.. R.L.ED..KI .NPRYQNQFA -E.H.R.S.H ..VV.AVD.K ..N.TCHL.D L.SK.K.PNN .RIHVGKVL- PKTG..MTF. VV..L..NC.
Hosa_NDN         .VQ.AH..MW YV.V.D.K.M I.WFPD.VKD ..-GS.KKWC RS.L.RTSLI LARV..LH.. LTSLHTMEFA L.KA.EPEEL DRVALSNR.- PMTG..LM.. .L.YV..RG.
Hosa_MAGED1      .Q.RANK.VK Y.M..DYT.V ..KRS...RD I.-RE..DVY .E.IER.CFV L.KK...Q.K .I.KEEHL.I LIS.PE-SLA .I.GTTKDT- PKLG..LV.. GV..MN.NR.
Hosa_MAGED2      .QGRAND.VK Y..A.D.T.I ..KRSD..KD I.-KE..DVY .E.IER.GYS L.KV...Q.K .I.KNDHL.I LLS..EP.DA .I.GTTKDS- PKLG..MVL. ....MN.NRS
Hosa_TRO         .Q.RANK.VK Y..V.D.T.I ..KRSD..RD ..-QE.DE.. .E.IER.SYT L.KM.RVN.K .I.KQSSL.I LIS.QE-S.A .I.GTTKDT- PKLG..MV.. .V..MN.NK.
Hosa_MAGED4      .Q.RANK.VK Y.MI.DYK.I ..KR.D..KD ..-RE.DEH. .E.IER.TYT L.KK...H.K .I.KEEHL.I L.C.R.-S.A RL.GKTKDT- PRLS..LV.. GV..MN.NR.
Hosa_MAGED4B     .Q.RANK.VK Y.MI.DYK.I ..KR.D..KD ..-RE.DEH. .E.IER.TYT L.KK...H.K .I.KEEHL.I L.C.R.-S.A RL.GKTKDT- PRLS..LV.. GV..MN.NR.
Hosa_MAGEE1      MEQN.A..LQ ...V.D.S.Y ..RES..-RE Y.VKE.RNQ. .E.L.R.AAH L.CI.RFE.. .L..EAHT.I LL.K.GPVPF EG.EESPNG- PKMG..MM.. GQ..LN.NQ.
Hosa_MAGEE1_2    .ES.ARK.VQ LF..MDST.L ..P.KGI.YY IG-RECSKV. .DLLNR.ART LNHVY.TE.V VL..RNH..T LY.RREMEET EEIV.SPN-R PG.NF.MQV. .F...M.NH.
Hosa_MAGEE2      .EDRSIA.VN .MRM.S.TEG S.QQS...-E FL-RE.SDQ. .E.L.R.SAH LDQV..LN.. VI..QA.T.N L.SKRGFQIT DRIAESLD.- PKAS..A.V. GH.LLN.NR.
Hosa_MAGEE2_2    MND.AND.VQ LAISVTEEML ..HQD.L.AH TG-KEFEDV. .N.LNR.TLI LDMFY.L..I ...TSEHI.L L.QQPESEE. QVMLESL.-R PTQEYVMP.. GL..LM.NRV
Hosa_MAGEF1      .NRT.A..VQ ...V.DKK.S ...RS..VKY ..-GDLKIL. .D.IAR.A.H LRYV..FE.K QF.RKHHT.I LI.K.KPLE. EEEE.LG.DG PRLG..MM.. GL.YMR.NS.
Hosa_MAGEL2      ...RANA.VQ ...V.D.A.V .VQRS..VKV IL-RE.KDEC LD.INR.NNK L.CA..YQ.K .I.TKNHA.I II.K.GYHTG NLVA-SYLDR PKFG..MVV. .L..M..NCV
Hosa_NDNL2       .EL..S..VQ ...I.D.K.I ..KR.DI.KH ..-GD.KDI. .DL.KR.A.R LQYV..YK.V .LE.KSNT.I LI...EPVE. DAEMRGDQGT PTTG..MIV. GL..M..NTI
```

	111	121	131	141	151	161	171	181	193
Hosa_MAGEC1	SEEVIWDVLS	GIGVRAGREH	F--AFGEPRE	LLTKVWVQEH	YLEYREVPNS	SPPRYEFLWG	PRAHSEVIKR	KVVEFLAMLK	NTV
Hosa_MAGEC2	......E..N	AV..Y.....	.--VY.....	........G.	........H.	...Y......	......S..K	..L....K.N	...
Hosa_MAGEC3	P.....E...	A...C.....	.--IY.D..K	...IH...RK	....	A.........	......AS..	SLRV.IQAIQ	YHP
Hosa_MAGEA1	P..E..EE..	VME.YD....	S--.Y....K	...QDL...K	.....Q..D.	D.A.......	...LA.TSYV	..L.YVIKVS	AR.
Hosa_MAGEA2	P..K..EE..	MLE.FE...D	S--V.AH..K	..MQDL...N	.....Q..G.	D.AC......	...LI.TSYV	..LHHTLKIG	GEP
Hosa_MAGEA3	P..K..EE..	VLE.FE...D	S--IL.D.KK	...QHF...N	.....Q..G.	D.AC......	...LV.TSYV	..LHHMVKIS	GGP
Hosa_MAGEA2B	P..K..EE..	MLE.FE...D	S--V.AH..K	..MQDL...N	.....Q..G.	D.AC......	...LI.TSYV	..LHHTLKIG	GEP
Hosa_MAGEA6	P..K..EE..	VLE.FE...D	S--I..D.KK	...QYF...N	.....Q..G.	D.AC......	...LI.TSYV	..LHHMVKIS	GGP
Hosa_MAGEA12	P..K..EE..	VLEASD...D	S--V.AH..K	...QDL...N	.....Q..G.	D.AC......	...LV.TSYV	..LHH.LKIS	GGP
Hosa_MAGEA8	P..A..EA..	VM.LYD....	S--VYWKL.K	...QE....N	.....QA.G.	D.V.......	...LA.TSYV	..L.HVVRVN	AR.
Hosa_MAGEA4	...E..EE.G	VM..YD....	T--VY....K	...QD....N	.....Q..G.	N.A.......	...LA.TSYV	..L.HVVRVN	AR.
Hosa_MAGEA9	P.....EA..	VM..YV.K..	M--FY....K	...QD....N	.....Q..G.	D.AH......	SK..A.TSYE	..INY.V..N	ARE
Hosa_MAGEA10	P.....EA.N	MM.LYD.M..	L--IY....K	...QD....N	.....Q..G.	D.A.......	....A.IR.M	SLLK...KVN	GSD
Hosa_MAGEA11	P...M.E...	IM..Y.....	.--L....KR	...QN....K	..V..Q..GT	D.AC......	....A.TS.M	..L.YI.NAN	GRD
Hosa_MAGEA9B	P.....EA..	VM..YV.K..	M--FY....K	...QD....N	.....Q..G.	D.AH......	SK..A.TSYE	..INY.V..N	ARE
Hosa_MAGEB1	T..E..KFMN	VL.AYD.E..	L--IY....K	FI.QDL...K	..K.EQ....	D....Q....	...YA.TT.M	..L....KMN	GAT
Hosa_MAGEB4	R..E..EF.N	ML.IYD.KR.	L--I.....K	.I.QDL...K	....QQ....	D....Q....	....A.TS.M	..L....KVN	D.T
Hosa_MAGEB3	T..K..EF.N	KMRIYD.KK.	.--I.....K	.I.QDL.KLK	.....Q....	N.A.......	....A.TS.M	..L..W.KVN	K..
Hosa_MAGEB2	T..E..EF.N	ML..YD.E..	S--V....WK	.I..DL...K	....KQ..S.	D...FQ....	...YA.TS.M	..L....KVN	G.T
Hosa_MAGEB6	T..EV.EF.G	LL.IYD.IL.	S--IY.DA.K	II.EDL..DK	.VV..Q.C..	D..C......	...YA.TT.M	R.LRV..DSS	..S
Hosa_MAGEB18	P..AV.EIMN	MM..Y.D.K.	.--LY.D..K	VM..DL..LK	....QQ....	D.........	....A.TS.M	..L..V.KIH	D..
Hosa_MAGEB16	T..EV.E..N	LT..YS.KK.	.--I.....M	.I..DF.K.K	....QQ.A..	D.A.......	...KA.TS.M	..L..V.KVH	GSY
Hosa_MAGEB10	A..EV.K.FN	TM.LYD.I..	.--M.....K	....DL.K.N	....QQ....	D....Q....	....A.TS.M	..L....KVN	D.A
Hosa_MAGEB17	T..EM.EC.N	AL.MYK..K.	.--IY...Q.	.V..DL.R.G	....QQ..S.	D.........	...RA.TS.M	..L..V.K.N	D..
Hosa_MAGEB5	NK.DT.KF.D	MMQIYD.KKY	Y--IY....K	.I.QDF.RLT	....HQ..C.	Y.AH.Q....	...YT.TS.M	..L.Y..KVN	DIA
Hosa_NDN	R.SAV.N..R	IL.L.PWKK.	S--T..DV.K	.I.EEF..MN	..K.QR..YV	E..E...F..	S..SR.IT.M	QIM....RVF	KKD
Hosa_MAGED1	..A.L.EA.R	KM.L.P.VR.	P--LL.DL.K	...YEF.KQK	..D..R....	N..E......	L.SYH.TS.M	..LR.I.EVQ	KRD
Hosa_MAGED2	..A...E..R	KL.L.P.IH.	S--L..DVKK	.I.DEF.KQK	..D.AR....	N..E...F..	L.SYY.TS.M	..LK.ACKVQ	KKD
Hosa_TRO	..A...E..R	KL.L.P.VR.	S--L...V.K	.I.DEF.KQK	....KR....	R..E...F..	L.SYH.TS.M	..LK.ACRVQ	KKD
Hosa_MAGED4	..A.L.EA.R	KM.L.P.VR.	P--FL.DL.K	.I.DDF.KQK	....KKI...	N..E......	L..RH.TS.M	R.LR.I.QNQ	.RD
Hosa_MAGED4B	..A.L.EA.R	KM.L.P.VR.	P--FL.DL.K	.I.DDF.KQK	....KKI...	N..E......	L..RH.TS.M	R.LR.I.QNQ	.RD
Hosa_MAGEE1	K.AE..EM.W	RM..--Q..R	RLSI..N.KR	..SVEF.WQR	..D..P.TDC	K.VE...F..	..S.L.TT.M	.ILK.M.KIY	.KD
Hosa_MAGEE1_2	R.SAV.AF.R	.L..------	---QA.-RKH	VI.CRYLSQR	.IDSLR..D.	D.VQ...V..	...RL.TS.M	.ALRYV.RIH	RKE
Hosa_MAGEE2	R.AS...L.L	KVDMWDKPQR	INNL..NT.N	...TDF.CMR	F...WP.YGT	N.LEF.....	S...R.IT.M	EALK.VSDAH	DEE
Hosa_MAGEE2_2	K.ANV.NL.R	RFS.DV..K.	S---------	-I.RKLMRQR	...C.PLSY.	N.VE..L...	....H.T..M	..L.YM.R.Y	RKR
Hosa_MAGEF1	R.AQV.EM.R	RL..QPSKY.	.--L..Y.KR	.IMEDF..QR	..S..R..HT	N..E...S..	..SNL.IS.M	E.LG.V.K.H	KKE
Hosa_MAGEL2	R.DL.FNF.F	KL.LDV-..T	NG-L..NTKK	.I.E.F.RQK	.....RI.YT	E.AE......	...FL.TS.M	L.LR...K.H	KKD
Hosa_NDNL2	K.TEA..F.R	RL..Y-PTKK	HL-I..D.KK	.I.EDF.RQR	.....RI.HT	D.VD...Q..	..TNL.TS.M	..LK.V.KVH	.QD
