# Supplementary material for: Evolutionary History of the Cancer Immunity Antigen MAGE Gene Family
Source: PLoS One. 2011 Jun 10;6(6):e20365. doi: 10.1371/journal.pone.0020365 (PMC3112145; doi:10.1371/journal.pone.0020365)
Supplement: Table S1 — Accession numbers of nulceotide sequences used in this study. Species names are shown in colored cells. (PDF) [file pone.0020365.s005.pdf]

Table S1. Accession numbers of nulceotide sequences used in this study.

humans		chimpanzees		mice		bovines	
MAGEA1	NM_004988	MAGEA1-like	XM_529226	MAGEA1	NM_020015	MAGEB5-like	XM_001251181
MAGEA2	NM_005361	MAGE-3	XM_001136905	MAGEA2	NM_020016	MAGEA9-like	XM_603753
MAGEA2B	NM_153488	MAGEA4-like	XM_521309	MAGEA3	NM_020017	MAGEA9B	XM_603753
MAGEA3	NM_005362	MAGEA8	XM_529192	MAGEA4	BC104089	MAGEA10-like	NC_007331
MAGEA4	NM_001011548	MAGE-9-like	XM_529190	MAGEA5	NM_020018	MAGEA11	NM_001080732
MAGEA5	NM_021049	MAGE-10	XM_521312	MAGEA6	NM_020019	MAGEB16-like	XM_586788
MAGEA6	NM_005363	MAGEA11	XM_521299	MAGEA7	XM_001481307	MAGEB2-like	XM_001789276
psMAGEA7	NG_001156	MAGEA12	XM_521314	MAGEA8	NM_020020	MAGEB3	XM_586930
MAGEA8	NM_005364	rhesus macaques		MAGEA9	BC116353	MAGEB4	XM_587577
MAGEA9	NM_005365	MAGE-3L	XM_001100355	MAGEA10	NM_001085506	MAGEB4-like	XM_001256490
MAGEA9B	NM_001080790	MAGEA3	XM_001094934	MAGEB1	NM_010759	MAGEB10	XM_608078
MAGEA10	NM_001011543	MAGEA3L	XM_001094083	MAGEB2	NM_031171	MAGEB18-like	XM_602824
MAGEA11	NM_005366	MAGE-4-like	XM_001099496	MAGEB3	NM_008545	MAGEE2	BT030739
MAGEA12	NM_005367	MAGEA9	XM_001089793	MAGEB4	NM_001033492	MAGEF1	NM_030801
psMAGEA	NC_000023	MAGEA10	XM_001099898	MAGEB5	BC116773	MAGEH1	NM_001080728
MAGEB1	NM_002363	MAGEA11-like	XM_001089907	psMAGEB7	NM_001101595	NDN	BT020845
MAGEB2	NM_002364	opposums		psMAGEB8	NM_001101541	NDNL2	NM_001078080
MAGEB3	NM_002365	MAGEL1	XM_001373641	MAGEB9	XM_141933	MAGEL2-like	XM_581873
MAGEB4	NM_001033492	MAGEL2	ENSMODT00000021264	MAGEB16	XM_135953	MAGED1	NM_001046125
MAGEB5	XM_293407	platypuses		MAGEB18	NM_173783	MAGED2	NM_001075665
MAGEB6	NM_173523	MAGEL1	XM_001510461	MAGEE1	NM_053201	MAGED4-like	NM_001103311
MAGEB10	NM_182506	zebrafishes		MAGEE2	BC138210	LOC781970	XM_001250528
MAGEB16	XM_001099921	NDNL2	NM_198812	MAGEH1	BC060080	LOC520085	XM_598316
MAGEB17	XM_001130425	guinea pigs		NDN	NM_010882	LOC526966	XM_605350
MAGEB18	NM_173699	MAGEC-like	ENSCPOT00000023751	NDNL2	NM_030801	LOC788387	XM_001255465
MAGEC1	NM_005462	fruit flies		MAGEL2	NM_019066	LOC781934	XM_001789822
MAGEC2	NG_015872	MAGE	NM_141445	MAGED1	NM_019791	LOC781489	XM_001249922
MAGEC3	NM_138702	*Arabidopsis thaliana*		MAGED3	NM_001002272	LOC613503	XM_001787851
MAGEE1	NM_020932	MAGE-8 antigen-related	NM_179416	2410003J06Rik	XM_135953	LOC789863	XM_001256490
MAGEE2	NM_138703	chickens		EG236892	XM_141934	LOC539215	XM_585695
MAGEF1	NM_022149	MAGE	NM_001105064	EG436212	XM_488356	LOC520262	XM_598498
MAGEH1	NM_014061	lancets		LOC100046994	XM_001477195	LOC788982	XM_001790105
NDN	NM_002487	LOC100181542	XM_200119518	LOC623523	XM_888000	LOC522603	XM_600888
NDNL2	NM_138704	tunicates		LOC100046560	XM_001475499	LOC788399	XM_001255474
MAGEL2	NM_019066	hypothetical protein	XM_002613563	LOC100039436	XM_001472784	LOC783975	XM_001252415
MAGED1	NM_001005333					LOC781486	XM_001789276
MAGED2	BC000304					LOC618806	XM_87112
MAGED3/TRO	NM_001039705					LOC782979	XM_001251628
MAGED4	NM_001098800					LOC788989	XM_001790109
MAGED4B	NM_030801					LOC789236	XM_001256044

Species names are shown in colared cells.
