# Supplementary material for: Evolutionary History of the Cancer Immunity Antigen MAGE Gene Family
Source: PLoS One. 2011 Jun 10;6(6):e20365. doi: 10.1371/journal.pone.0020365 (PMC3112145; doi:10.1371/journal.pone.0020365)
Supplement: Table S2 — Nucleotide divergence among six MAGE-A genes from humans and macaques. Synonymous nucleotide divergences (below diagonal) and synonymous nucleotide divergences with removal of CG codons (upper diagonal) for the six MAGE-A genes were showed. Standard errors are provided in parentheses. Sequences are from humans (Hosa) and macaques (Mamu). The number of synonymous sites with CG codons is 226 and that without CG codons is 173. (PDF) [file pone.0020365.s006.pdf]

Table S2

Nucleotide divergence among six *MAGE-A* genes from humans and macaques.

	Hosa A4	*Hosa A3 (h6)*	*Hosa A6 (h2)*	*Mamu A3 (m6)*	*Mamu A3L (m4)*	*Mamu 3L (m2)*
Hosa A4		0.12 (0.024)	0.121 (0.024)	0.146 (0.026)	0.154 (0.027)	0.147 (0.026)
Hosa A3	0.158 (0.023)		0 (0)	0.064 (0.018)	0.093 (0.024)	0.076 (0.02)
Hosa A6	0.155 (0.023)	0.018 (0.008)		0.064 (0.018)	0.093 (0.024)	0.076 (0.02)
Mamu A3	0.211 (0.026)	0.139 (0.024)	0.129 (0.023)		0.058 (0.019)	0.023 (0.011)
Mamu A3L	0.195 (0.026)	0.142 (0.023)	0.133 (0.022)	0.062 (0.016)		0.058 (0.017)
Mamu 3L	0.193 (0.025)	0.125 (0.022)	0.116 (0.020)	0.063 (0.017)	0.036 (0.012)	

Synonymous nucleotide divergences (below diagonal) and synonymous nucleotide divergences with removal of CG codons (upper diagonal) for the six *MAGE-A* genes. Standard errors are provided in parentheses. Sequences are from humans (Hosa) and macaques (Mamu). The number of synonymous sites with CG codons is 226 and that without CG codons is 173.
